# Stepwise target controllability identifies dysregulations of macrophage networks in multiple sclerosis

**DOI:** 10.1162/netn_a_00180

**Published:** 2021-04-27

**Authors:** Giulia Bassignana, Jennifer Fransson, Vincent Henry, Olivier Colliot, Violetta Zujovic, Fabrizio De Vico Fallani

**Affiliations:** Sorbonne University, UPMC Univ Paris 06, Inserm U-1127, CNRS UMR-7225, Institut du Cerveau et de la Moelle Epinière, Hopital Pitié-Salpêtrière, Paris, France; Inria Paris, Aramis Project Team, Paris, France; Sorbonne University, UPMC Univ Paris 06, Inserm U-1127, CNRS UMR-7225, Institut du Cerveau et de la Moelle Epinière, Hopital Pitié-Salpêtrière, Paris, France; Sorbonne University, UPMC Univ Paris 06, Inserm U-1127, CNRS UMR-7225, Institut du Cerveau et de la Moelle Epinière, Hopital Pitié-Salpêtrière, Paris, France; Inria Paris, Aramis Project Team, Paris, France; Sorbonne University, UPMC Univ Paris 06, Inserm U-1127, CNRS UMR-7225, Institut du Cerveau et de la Moelle Epinière, Hopital Pitié-Salpêtrière, Paris, France; Inria Paris, Aramis Project Team, Paris, France; Sorbonne University, UPMC Univ Paris 06, Inserm U-1127, CNRS UMR-7225, Institut du Cerveau et de la Moelle Epinière, Hopital Pitié-Salpêtrière, Paris, France; Sorbonne University, UPMC Univ Paris 06, Inserm U-1127, CNRS UMR-7225, Institut du Cerveau et de la Moelle Epinière, Hopital Pitié-Salpêtrière, Paris, France; Inria Paris, Aramis Project Team, Paris, France

**Keywords:** Network controllability, Molecular networks, Multiple sclerosis, Neural disease

## Abstract

Identifying the nodes able to drive the state of a network is crucial to understand, and eventually control, biological systems. Despite recent advances, such identification remains difficult because of the huge number of equivalent controllable configurations, even in relatively simple networks. Based on the evidence that in many applications it is essential to test the ability of individual nodes to control a specific target subset, we develop a fast and principled method to identify controllable driver-target configurations in sparse and directed networks. We demonstrate our approach on simulated networks and experimental gene networks to characterize macrophage dysregulation in human subjects with multiple sclerosis.

## Author Summary

We introduce an optimized heuristic, called stepwise target controllability, to quantify the centrality of a candidate driver node to influence the state of a network target set. We use this method to study macrophage gene network alterations in multiple sclerosis. We show that multiple sclerosis is characterized by a global loss of gene coactivation and that this is due to the dysregulation of few molecules along the driver-target pathways. These findings provide new insights into the macrophage network mechanisms underlying the pathophysiology of multiple sclerosis and provide fresh tools for the study of driver-target controllability in complex networked systems.

## INTRODUCTION

For many biological systems, it is crucial to identify the units, such as genes or neurons, with the potential to influence the rest of the network, as this identification can enable describing, understanding, and eventually controlling the function of the system (Bonifazi et al., 2009; Jeong, Mason, Barabási, & Oltvai, 2001). Topological descriptors based on network science can indeed be used to quantify such influence in terms of node centrality, such as degree, betweenness, or closeness (Newman, 2010). However, these descriptors capture only the structural properties of the network and neglect their effect on the dynamics, thus limiting our understanding on the actual influencing power.

Control network theory, linking network structure to dynamics through linear or nonlinear models, has been shown to be a more principled approach for identifying driver nodes in an interconnected system (Rugh & Kailath, 1995; Sontag, 1998). While theoretically these approaches can give a minimum set of driver nodes sufficient to steer the system into desired states, their exhaustive identification might be difficult in practice as there exists in general a very large number of equivalent controllable walks, even in relatively simple networks (Heuberger & Wagner, 2011; S. G. Wagner, 2007). In the case of criteria based on the manipulation of controllability matrices (Hautus, 1970; Kalman, 1963), the presence of many walks can for example induce numerical errors due to the different orders of magnitude in the matrix elements.

An alternative solution has recently been proposed to circumvent this limitation, based on the possibility to map the controllability problem onto the maximum cardinality matching over the associated graph (Lin, 1974; Y.-Y. Liu, Slotine, & Barabási, 2011; Shields & Pearson, 1976). As a result, it is possible to identify a set of driver nodes—at least for directed networks—with linear, and not exponential, time complexity (Hopcroft & Karp, 1971). While this approach elegantly solves numerical issues, it can nevertheless not tell which configuration, among all the possible ones, is the most relevant. In general, there is a factorial number of equivalent configurations (with the same number of inputs), and enumerating all possible matchings (Uno, 1997) rapidly becomes unfeasible, even for simple graphs such as trees (Heuberger & Wagner, 2011; S. G. Wagner, 2007), bipartite graphs (Y. Liu & Liu, 2004), or random graphs (Zdeborová & Mézard, 2006). Thus, the research of alternative strategies to characterize the candidate driver nodes is crucial for the concrete application of network controllability tools.

However, they do not solve the problem of multiple driver set configurations. On the other hand, technical and experimental constraints often limit the possibility to stimulate many driver nodes in parallel, for example in gene expression modulation (Lodish et al., 2000) or brain stimulation (Hallett, 2000). In these cases, approaches that focus on the ability of single driver node to control the entire network, such as control centrality (Y.-Y. Liu, Slotine, & Barabási, 2012) or single-node controllability (Gu et al., 2015), do circumvent the multiplicity issue, but can still suffer from numerical errors and approximate results.

To overcome this impasse, we propose an integrated method that combines the advantages of the previous approaches and quantifies the capacity of a single driver node to control a predefined target set. Based on the Kalman controllability condition, our method identifies the part of the target set that can be controlled by a candidate driver. To do so, we introduce a ranking among the target nodes and we iteratively evaluate the controllability of the system by adding one target node at a time in a descending order. This eventually finds a univocal controllable configuration corresponding to the highest ranking. In the following, we first illustrate how our method, named *stepwise target controllability*, works for simple network structures and we discuss the potential benefits for directed and sparse networks as compared with alternative approaches. Then, we use it to study molecular networks of macrophage pro-inflammatory activation, derived from ontology-based reconstructions, and identify the driver-target pathway alterations using gene expression data from blood samples of patients affected by multiple sclerosis (MS) and a matched group of healthy controls (HC).

## RESULTS

### Stepwise Target Controllability Identifies a Controllable Subset of Targets

Let 𝒢 be a directed graph (or network) of *N* nodes (or vertices) and *L* links (or edges), and 𝒯 an arbitrary subset of *S* < *N* nodes in the network. The aim is to measure the ability of each node to drive the state of the target set 𝒯> from a dynamical system perspective (Sontag, 1998). In the case of linear time-invariant dynamics, the number of controllable target nodes can be obtained by computing the rank of the target controllability matrix (Commault, Van der Woude, & Frasca, 2019; Gao, Liu, D’Souza, & Barabási, 2014; Murota & Poljak, 1990):

$$Q_{𝒯}=\left[CB\;CAB\;CA^{2}B\;\cdots \;CA^{N-1}B\right],$$ (1)

where *A* is the adjacency matrix of the network, *B* is a vector identifying the driver node, and *C* is a matrix selecting the rows of *A* corresponding to the target, or output, nodes (Material and Methods).

A *walk* in the network consists of an alternating sequence of vertices and edges.

The (*i*, *j*) entry of *Q*_𝒯_ indicates how many walks of length *j* − 1 connect the driver to the target *i* (Biggs, 1974). Trivially, all the nodes not traversed by these walks do not contribute to the walk lengths and they can be neglected for the purpose of control. By removing the irrelevant nodes from the network, *A* becomes smaller and this results in a target controllability matrix with fewer columns. Put differently, we avoid the computation of matrix exponentials corresponding to nonexisting driver-target walks (Material and Methods). In practice, this can be of great advantage for reducing the occurrence of round-off errors during the matrix rank calculations. For example, this is the case for sparse and directed networks, where fewer nodes are reachable as compared with dense and undirected networks.

This can be easily appreciated in the following example. Let us consider a directed full binary tree with *h* = 6 levels, with the root node as the candidate driver. Without loss of generality, we randomly position a target in each level and we rank them according to their height in the tree. Then, we introduce a simple cycle among the first three nodes of the tree (Figure S1 in the Supporting Information). By construction, this configuration is controllable and the entire target set can be fully driven by the driver. However, when considering the entire network the returned rank is deficient. Instead, by removing the part of the network that is irrelevant for the control, the rank is full and we retrieve the entire controllable configuration, even in the case of larger networks, that is, up to *h* = 10 levels.

**Figure 1.**
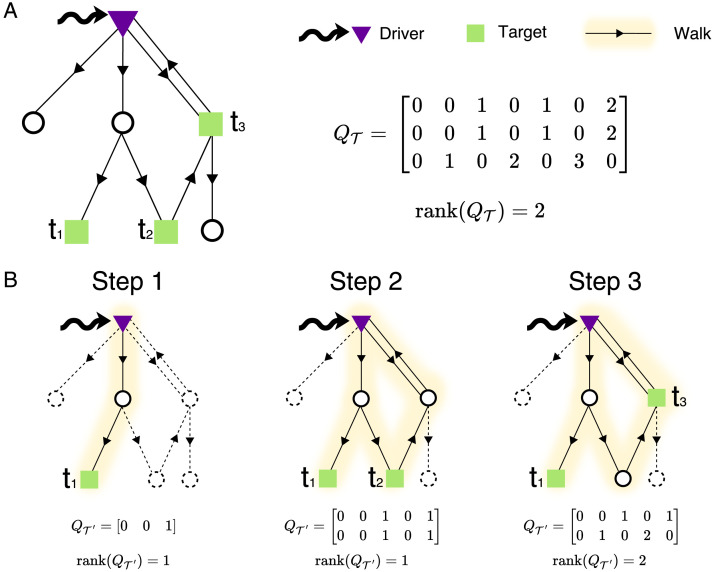
Working principle of stepwise target controllability. (A) A network with one driver and a target set 𝒯 = {*t*_1_, *t*_2_, *t*_3_} of cardinality *S* = 3. The Kalman condition informs us that only two targets are controllable from the driver, that is, *τ* = rank(*Q*_𝒯_) = 2. However, there might be up to three equivalent configurations that are controllable, that is, {*t*_1_, *t*_2_}, {*t*_1_, *t*_3_}, and {*t*_2_, *t*_3_}. For larger networks, the number of Kalman tests to perform can be prohibitive, that is, $\left(\begin{array}{c} S\\ \tau \end{array}\right)$. (B) By introducing a hierarchy among the target nodes, our stepwise method identifies the configuration with the most important nodes by performing only *S* tests (see Material and Methods). In this example, the first step considers the subgraph containing all the walks from the driver to the target set 𝒯′ = {*t*_1_}. The associated controllability matrix has full rank, that is, rank(*Q*_𝒯′_) = 1. The first target is therefore retained and the algorithm moves to Step 2, by constructing a new subgraph containing the walks from the driver to the target set 𝒯′ = {*t*_1_, *t*_2_}. The rank of the new controllability matrix is now deficient and *t*_2_ is not retained. In Step 3, the new subgraph contains the walks from the driver to 𝒯′ = {*t*_1_, *t*_3_}. Because rank(*Q*_𝒯′_) is full and there are no more targets, the algorithm stops and returns the controllable configuration *t*_1_, *t*_3_.

The rank of *Q*_𝒯_ gives the number of target nodes *τ* ≤ *S* controllable by the driver, but there might be in general many possible equivalent configurations. To overcome this issue, we propose a stepwise procedure that tests the controllability on subproblems of increasing size. First, we introduce a hierarchy among the target nodes and relabel them according to their importance in a descending order, that is, *t*_1_ ≻ *t*_2_ ≻ … ≻ *t*_*S*_. Then, we create an empty auxiliary set 𝒯′ and we sequentially include the target nodes according to their ordering. At each step, if the rank of *Q*_𝒯′_ is full, the new target node is retained, otherwise it is removed from 𝒯′. When all the target nodes have been visited, the algorithm returns the set of controllable targets with highest ranking (Figure 1; Material and Methods).

Our method, named *stepwise target controllability*, returns for each candidate driver not only the number of controllable target nodes *τ* corresponding to the configuration with highest ranking, but also the set 𝒯′ of controllable targets.

To explore the limits of our method in terms of computational complexity, we performed a simulation analysis on synthetic random networks, which vary in number of nodes, connection density, and target size (Figure S2 in the Supporting Information). Results show that in general our method is able to retrieve a larger number of controllable targets as compared with a standard approach that computes the rank of the full controllability matrix. More specifically, when the target set contains 5% of the network nodes, results are quite stable across different connection densities. For larger target-set sizes, our method works better when the connection density is relatively low (0.02–0.10). It is important also to notice that the computation of the rank starts to fail in correspondence with larger and denser networks (i.e., *N* > 180 and density > 0.20).

### Driver Genes Are Homogeneously Distributed in the Macrophage Network

**Figure 2.**
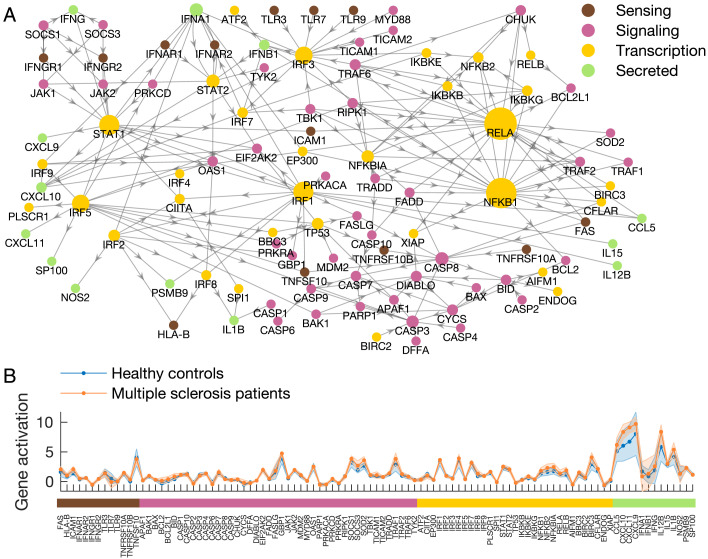
Molecular network and gene activation associated with the pro-inflammatory state of macrophages. (A) The molecular network reconstructed through ontology-based techniques from the macrophage.com repository (Raza et al., 2008; Robert, Lu, Law, Freeman, & Hume, 2011). The network consists of *N* = 101 nodes corresponding to genes involved in inflammation; for the sake of interpretablity, they are organized in four classes, depending on their function in the cell. *Sensing* genes are in the membrane of the cell and start a *signaling* pathway inside the cell, to the *transcription* factors, which promote the production of *secreted* molecules. There are *L* = 211 directed edges representing either activation or inhibition interactions between molecules (Material and Methods). The size of the nodes is proportional to their total degree *k*. (B) Gene activation computed as the ratio in expression between the “pro-inflammatory” and “alert” states, based on our RNA sequencing data, generated from monocyte-derived macrophages from blood samples of multiple sclerosis patients (*n* = 8) and healthy controls (*n* = 8) (Material and Methods). Solid lines represent group-averaged values, while transparent patches stand for standard deviation.

To test our method in a biological context, we construct a network representing the interactions between molecules involved in macrophages response to pro-inflammatory stimuli (Figure 2), with the connections between genes inferred from a previously established network based on literature (Raza et al., 2008; Robert, Lu, Law, Freeman, & Hume, 2011). This network is of interest in MS because of the chronic inflammation characteristic of the disease, and the generally destructive effects of pro-inflammatory macrophages in MS (Bitsch et al., 2000). Hence, dysregulation of macrophages may lead to aggravated inflammation and disease.

In order to facilitate biological interpretation of the network, we divide the nodes according to molecular function: *sensing*, *signaling*, *transcription* factors, or *secreted* molecules (Table S1). We choose the 13 *secreted* molecules as target nodes because they represent the end products of macrophage pro-inflammatory activation and enable propagation of inflammation to other cells, thus exacerbating chronic inflammation.

To establish a hierarchy among the targets, we use macrophage RNA expression data from a group of MS patients and healthy controls. The macrophages were tested with and without activating stimuli to mimic the pro-inflammatory response. We measure the gene activation as the ratio of the expression between the “pro-inflammatory” and “alert” condition. We then consider the fold change Δ between the gene activation of MS patients and HC subjects (Material and Methods). Genes with larger *Δ* values are ranked first (Figure S3 in the Supporting Information).

**Figure 3.**
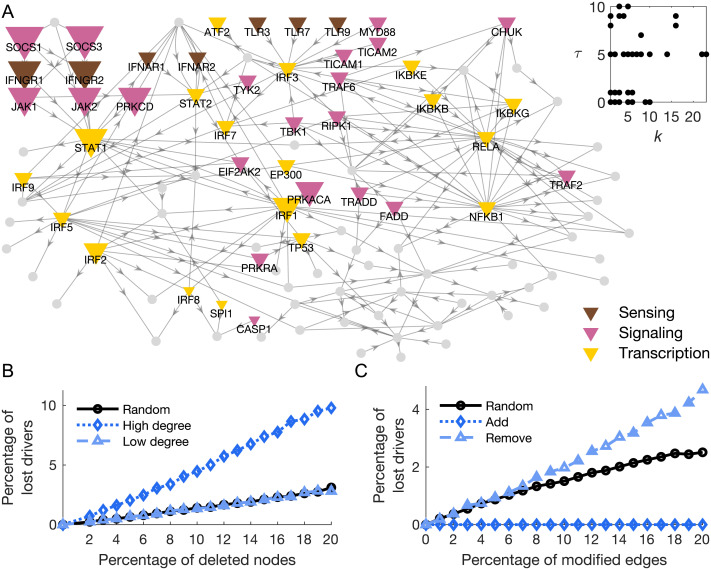
Gene network target control centrality and analysis of robustness for the driver nodes. (A) The size of the nodes codes the stepwise target control centrality values *τ*. Nodes with *τ* = 0 are classified as not-drivers and are represented in gray. The inset shows that *τ* values cannot be merely predicted by node degree *k* (Spearman rho 0.18, *p* < 0.07). (B) The percentage of driver nodes (*τ* > 0) that are lost when removing nodes in a random fashion (black circles), or preferentially attacking high-degree (blue diamonds) or low-degree nodes (light blue triangles). (C) The percentage of driver nodes that are lost when randomly rewiring (black circles), adding (blue diamonds), or removing edges (light blue triangles).

Results show that 51% of the tested network nodes can control at least one target (i.e., *τ* > 0) and that those drivers tend to be homogeneously distributed across classes (Table S2). This indicates a high redundancy in the way the target set can be controlled. Notably, target centrality values are weakly correlated (Spearman rho 0.18, *p* < 0.07) with the corresponding total node degree *k*, as defined in Material and Methods, indicating that the most connected genes (e.g., RELA, NFKB1) are not necessarily the ones that can most efficiently steer the state of the target set (Figure 3A).

Almost all of the driver nodes identified by our method can control the target genes CCL5, CXCL10, CXCL11, IFNA1, and IFNB1, which code for inflammatory chemokines and cytokines. This implies a high level of coregulation among these molecules, with many different actors exerting control over this regulation. Interestingly, the drivers with the highest target control centrality values (SOCS1 and SOCS3, *τ* = 10) belong to feedback systems that control pro- and anti-inflammatory signal transduction by regulating the signaling process triggered in response to IFN*γ* (McCormick & Heller, 2015). In addition, all drivers with *τ* ≽ 9 can be seen in our network as a cluster of genes converging onto and including STAT1 (Figure 3A). This cluster includes the receptors of IFN*γ* and the signaling molecules responsible for their intracellular effects. This result matches the well-described effects of IFN*γ* on chemokine production (Koper, Kamińska, Sawicki, & Kemona, 2018; J. Liu, Guan, & Ma, 2005) and overall macrophage activation (Mosser & Edwards, 2008).

#### Robustness of driver nodes to random attacks.

To assess the stability of our findings to possible errors in the network construction, we performed a robustness analysis simulating different types of alterations to its nodes and links (Material and Methods). Results show that removing nodes with higher degree *k* leads to a greater reduction of control centrality in the drivers compared with the removal of low-degree nodes or random removal of nodes (Figure 3B). For example, by attacking 10% of the nodes we lose 5% of the drivers in the latter cases, while we lose 20% of the drivers when removing the most connected ones (Figure 3B). This result confirms the crucial role of hubs in biological networks in terms of resilience to random attacks (Barabási & Oltvai, 2004) and controllability (Pu, Pei, & Michaelson, 2012).

When perturbing links, the worst condition is given by their random removal. By attacking 10% of the links, around 5% of the drivers are lost. This is intuitively due to the interruption of driver-target walks and to consequent impossibility to control a node that cannot be reached. While randomly rewiring the links has an intermediate impact, adding new links has no effect on the target control centrality of the drivers (Figure 3C). This is of great advantage as it shows that our results will not change if new connections are established or provided by the literature.

### Gene Dysregulation and Altered Driver-Target Coactivation in Multiple Sclerosis

**Figure 4.**
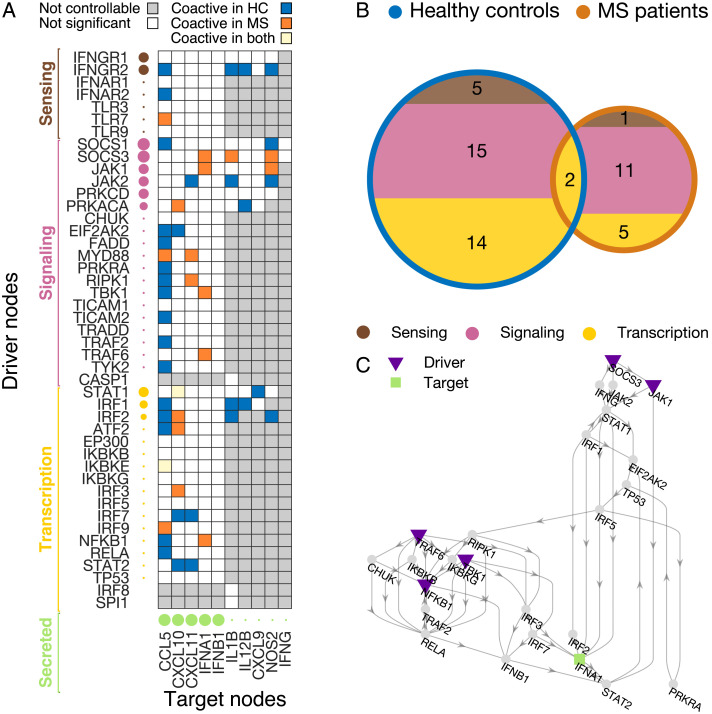
Altered driver-target coactivation in multiple sclerosis. (A) The coactive driver-target pairs, computed as significant Spearman correlations (*p* < 0.05) between the gene activation of controllable driver-target pairs, for the healthy control (HC - blue squares) and the multiple sclerosis group (MS - red squares). White squares indicate that there is a controllable walk from the driver to the target, but that their correlation is not significant. Gray squares mean that there is no controllable walk for driver-target pairs. The size of the circles for driver nodes codes for their target control centrality values *τ*. For target genes, circle sizes represent the number of driver nodes that can control them. (B) Venn diagram showing a decrease in number of driver-target coactivations in the MS patients as compared with HC. In both groups, these functional interactions tend to predominantly involve signaling genes. (C) Subnetwork of the walks from all the drivers coactivated with the target IFNA1.

Using stepwise target controllability, we detect potential directed interactions in the macrophage activation network, but we cannot quantify how changes in the driver’s state affect those in the targets. To measure driver-target functional interactions, we compute the Spearman correlation between the gene activation of controllable driver-target pairs, for the HC and MS groups (Material and Methods, Table S3). We call *coactivated* the genes exhibiting a significant correlation (*p* < 0.05). Results show that in general only a moderate fraction (21%) of all the possible driver-target genes are coactivated (Figure 4A, Table S3). For both HC and MS groups these interactions tend to primarily involve signaling functions Figure 4B. However, the number of driver-target coactivations is lower in the pathological condition (*MS* = 19 versus *HC* = 36). More importantly, they differ from those observed in the HC group (Figure 4B). This is particularly evident for target IFNA1, which only exhibits coactivations with signaling and transcription drivers in the MS group (Figure 4C).

**Figure 5.**
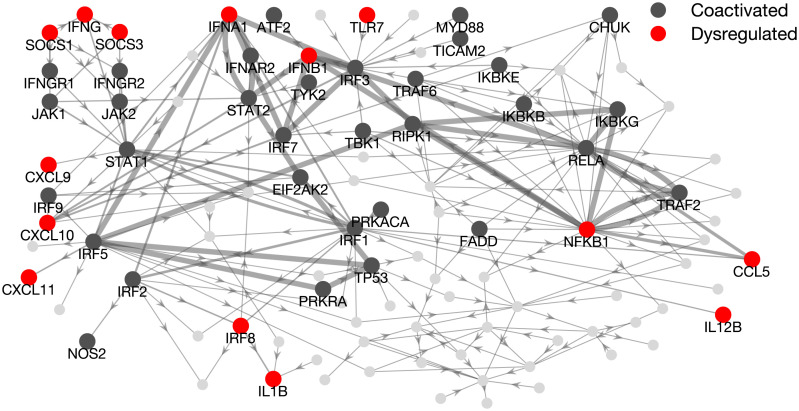
Pooled visualization of dysregulated genes along differentially coactivated driver-target walks. Highlighted genes indicate all nodes on walks between coactivated driver-target pairs, either in the healthy control group, or in the MS patients group. Dysregulated genes are shown in red. Edge thickness is proportional to the number of times they are traversed by walks connecting a driver to a target node (information not reported here).

Because the macrophage network edges are fixed and reconstructed from known protein-protein interactions, differences in coactivation can be essentially attributed to altered regulation of transcription. Hence, our hypothesis is that the observed functional reorganization can be explained by the dysregulation of specific genes along the controllable walks from the drivers to targets. To test this prediction, we examine all the pairs of genes whose coactivation appears or disappears in the MS group (Figure 4A). We found that 47/51 of these differentially coactivated pairs present at least one dysregulated gene (i.e., fold change |Δ| above the 75th percentile, Material and Methods) on the walk from the driver to the target (Figure 5, Table S4).

We find in total 14 dysregulated nodes on any of these walks. The genes that most frequently appear are NFKB1, IFNA1, and IFNB1 (36/51 walks). They are present on all walks that end with targets CCL5, CXCL10, CXCL11, IFNA1, or IFNB1, that is, the five targets that could be controlled by most drivers. This points to their dysregulation being a potent disruptor of the normal network functioning. The co-occurrence of these three dysregulated genes can be explained by a feedback loop in which NFKB1 activates IFNB1, and IFNA1 and IFBN1 both activate STAT2, which through several intermediates can influence all three genes (Figure S4 in the Supporting Information). Indeed, this stems from the fact that all these nodes belong to the main connected component of the network, that is a subnetwork in which every node is reachable from any other node.

Taken together, these results indicate that the aberrant reorganization of functional interactions in the MS group is associated with the presence of dysregulated genes along the controllable walks of the macrophage network.

#### Switch of SOCS-gene coactive drivers reflects dysregulated inflammatory response.

**Figure 6.**
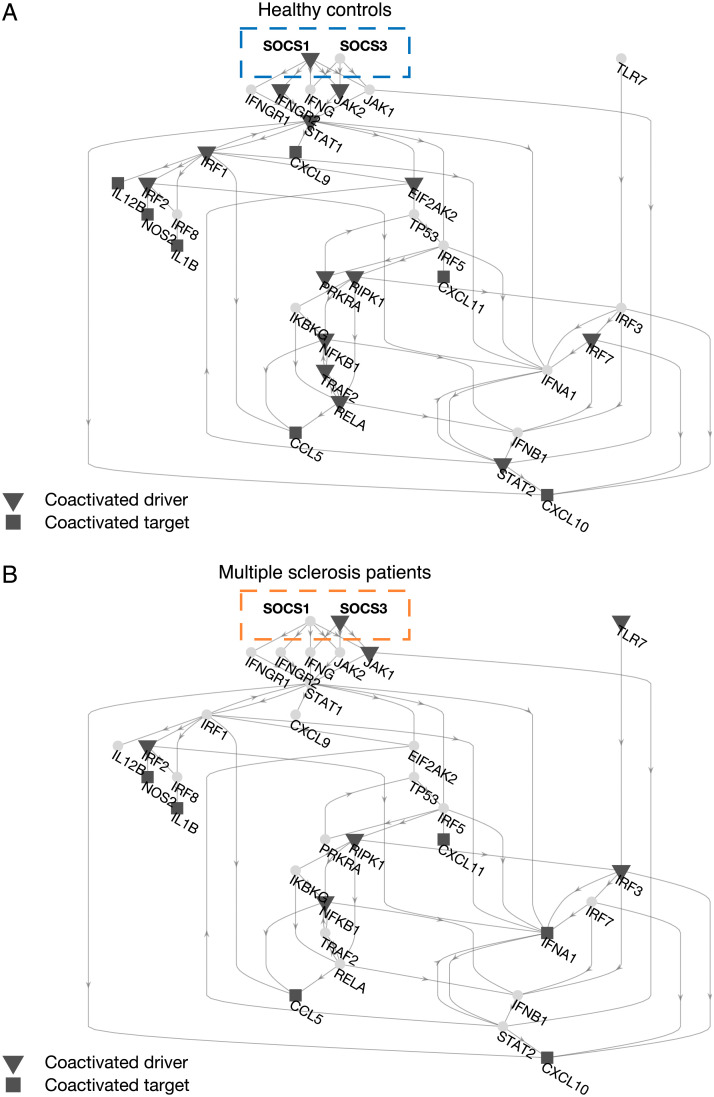
Dyregulated drivers and coactivation switch for SOCS-genes. The subnetwork includes all dysregulated drivers (IRF8, NFKB1, SOCS1, SOCS3, TLR7) and their controllable targets. (A) Coactivated pairs for healthy controls (HC). (B) Coactivated pairs for multiple sclerosis (MS) patients. A coactivation switch can be appreciated between the HC and MS group. SOCS1 and SOCS3 are respectively coactive and silent in HC, while they invert their role in the MS group.

Because drivers are crucial for steering the target network’s state, we focus on the subnetwork specifically involving the dysregulated drivers (IRF8, NFKB1, SOCS1, SOCS3, TLR7) and the walks towards the respective controllable targets (Figure 6). By looking at how driver and target nodes are differently coactivated in healthy controls and MS patients, we obtain a much clearer description of the gene dysregulation effects. First, many of the previous results can be now appreciated in finer detail, such as (a) the reduction in number of coactivated driver-target pairs in MS, (b) the large number of targets that can be controlled by SOCS1 and SOCS3, and (c) the potential of NFKB1, IFNA1, and IFNB1 to affect the driver-target functional interactions.

Second, we report an interesting mechanism involving the drivers with the highest *τ* centrality values, that is, SOCS1 and SOCS3. In the HC group, SOCS1 is coactivated with the targets while SOCS3 does not exhibit any significant correlation. In the MS group, we observe the opposite, that is, SOCS1 is silent while SOCS3 becomes coactive. Because both driver genes are dysregulated, the observed “switch” mechanism could therefore be associated with the altered pro-inflammatory response of the MS group. Indeed, these two molecules are known to be strong modulators of macrophage response: SOCS1 inhibits the signaling of pro-inflammatory genes, while SOCS3 is known to be an important actor in inflammatory response, with the ratio of the two proteins determining the actual effect (Wilson, 2014).

## DISCUSSION

### Identification of Controllable Configurations in Complex Networks

Network controllability refers to the ability to drive an interconnected dynamical system from any initial state to any desired final state in finite time, through a suitable selection of inputs (Rugh & Kailath, 1995; Sontag, 1998). In recent years, an increasing number of research groups from different disciplines have focused their efforts on identifying the minimum set of driver nodes or quantifying the capacity of single nodes to control the entire network, as well as parts of it (Betzel, Gu, Medaglia, Pasqualetti, & Bassett, 2016; Dosenbach et al., 2007; Gu et al., 2017; Y.-Y. Liu & Barabási, 2016; Lugagne et al., 2017; Menara, Bassett, & Pasqualetti, 2019; Muldoon et al., 2016; Sun et al., 2016; Tang et al., 2017; T. Wagner, Valero-Cabre, & Pascual-Leone, 2007; Wuchty, 2014). Despite being theoretically attractive, network controllability still suffers from computational issues that limit its impact in concrete applications. This is mainly due to the presence of multiple equivalent controllable walks in a network that make the associated controllability problem ill-posed and/or the resulting solution space very big (Rugh & Kailath, 1995; Sontag, 1998).

To reduce such complexity, we propose a method based on control centrality, which was previously designed to quantify the ability of one node to control directed networks (Y.-Y. Liu et al., 2012). First, we define the *target* control centrality to measure the controllability of a specific part of the network, that is, a predefined target set. Because edges are directed, this has the advantage of ignoring the part of the network that is not traversed by the walks connecting the driver to the target set. Second, we introduce an ordering among the target nodes and perform a stepwise controllability test with increasing size. Because of the ranking, only one controllable configuration will be identified, that is, the one with the highest ranking (Figure 1). To test the controllability of the driver-targets configuration at each step, we adopted the Kalman criterion (Kalman, 1963; Rugh & Kailath, 1995). However, the entire iterative framework is quite flexible and other methods, such as Gramian condition (Klickstein, Shirin, & Sorrentino, 2017), Popov-Belevich-Hautus criterion (J. Li, Chen, Pequito, Pappas, & Preciado, 2018), or feedback vertex set (Zañudo, Yang, & Albert, 2017), could be used as alternative controllability criteria.

The main advantage in terms of time of our method is that we identify a controllable driver-targets configuration by performing *j* tests when *j* < *S* out of *S* target nodes are controllable, as opposed to a brute-force approach that requires us to test all the possible configurations with *j* targets and that, in the worst-case scenario, leads to $\left(\begin{array}{c} j\\ S \end{array}\right)$ tests.

In addition, because of the stepwise procedure, the method does not need to compute the rank of the full controllability matrix and this allows us to minimize possible round-off errors, at least for relatively small networks. Specifically, our results show that the computations are reliable when *N* < 180 and the connection density is < 0.2 (Figure S2). While these numbers are in general relatively low and impede to scale up to very large networks, they still represent ranges that are compatible with typical brain networks obtained from neuroimaging data (E. Bullmore & Sporns, 2009; E. T. Bullmore & Bassett, 2011; De Vico Fallani, Richiardi, Chavez, & Achard, 2014).

### Control Pathways in Macrophage Molecular Networks

The study of the molecular interactions is crucial to the understanding of the basic functions of the cell such as proliferation or apoptosis (Maslov & Sneppen, 2002; Taylor et al., 2009). Determining the connection mechanisms that rule a specific biological function can significantly impact our daily life by providing new therapeutics to counteract diseases (Drier, Sheffer, & Domany, 2013; Menche et al., 2015; Tong et al., 2017). Studying molecular networks is however difficult, because in general we do not know the true functional interactions of a cell and indirect techniques such as gene coexpression are typically employed to infer such connections (Uygun, Peng, Lehti-Shiu, Last, & Shiu, 2016). Based on correlation analysis, these methods cannot inform us about the causal nature of the interactions. More importantly, the reliability of the estimated network critically depends on the number of interactions to number of data samples ratio, which is in practice very low (Uygun et al., 2016).

To overcome this limitation, we reconstruct the directed gene interactions associated with the inflammatory state of the human macrophages by adopting a novel ontology-based approach that integrates the available information from multiple datasets and results in the literature (Henry et al., 2017). Previous studies show that the number of driver nodes in biological networks is rather high because of their sparse and heterogeneous nature (Y.-Y. Liu et al., 2011; Ruths & Ruths, 2014). Consistently, we find that a large percentage of genes (51%) can control at least one secreted molecule in the target set. Our results also confirm that, despite being crucial for global communication, hubs (e.g., RELA) are not always the most important from a network control perspective (Figure 3A). This stems from the theoretical impossibility to diversify the input signals to all the connected neighbors (Y.-Y. Liu et al., 2011). The found driver genes are heterogeneously distributed across the tested gene classes. However, our method highlights SOCS1 and SOCS3 as the drivers with the highest target control centrality values, with other IFN*γ*-response-related genes showing similar values. This is in line with the known effects of SOCS-genes and IFN*γ* on molecules secreted by pro-inflammatory macrophages (Koper et al., 2018; J. Liu et al., 2005; McCormick & Heller, 2015; Mosser & Edwards, 2008), supporting the ability of this method to identify biologically relevant drivers.

Overall, these results uncover the existence of potential causal influences from candidate driver genes to the secreted molecules in the human macrophage activation network. Because the identified driver nodes are robust to network alterations, notably when adding new links (Figure 3C, blue diamonds), the obtained results are expected to be sufficiently resilient to the integration of new gene-gene interactions. Notably, our results are also relatively robust to the deletion of links, a situation that is often associated with filtering or thresholding out the weakest or less relevant connections in biological networks (De Vico Fallani, Latora, & Chavez, 2017) (Figure 3C, light blue triangles).

From a different angle, our approach can be seen as a principled manner to focus on specific nodes (the drivers) or node pairs (driver-target) in complex networks. This might have important consequences when studying genome-wide databases where the high number of elements can make prohibitive the assessment of significant gene expressions and/or coexpressions (Bentley et al., 2008; Mullighan et al., 2007).

### Dysregulated Genes and Aberrant Interactions in Multiple Sclerosis

Multiple sclerosis is an immune-mediated disease in which the immune system erroneously attacks myelin in the central nervous system. There are many neurological symptoms, including motor and cognitive deficits, that can vary in type and severity depending on the attacked central nervous system regions (Hauser, Oksenberg, & Baranzini, 2015). The role of macrophages in MS is crucial because of their ability to obtain a pro-inflammatory activation state, including the release of pro-inflammatory cytokines and leading to central nervous system tissue damage (Chu et al., 2018). Hence, dysregulation of macrophages may lead to autoimmunity and persistent inflammatory diseases (Strauss, Dunbar, Bartlett, & Phillips, 2015). While the etiology of MS is still not well understood there is a large consensus on its genetic basis and on the importance of unveiling the underlying network mechanisms (Airas, 2015).

In this study, we combined network controllability tools and gene expression data to detect the genes responsible for altering the macrophage action in multiple sclerosis. Different from standard approaches, where the attention is focused on the identification of the driver nodes in a network, we here propose an alternative way of exploiting network controllability. We first show that the macrophage inflammatory state in the MS group was characterized by a drastic alteration of the coactivations in the driver and target genes (Figure 4). Such absence of coordination was in general associated with the presence of dysregulated genes along the walks from the driver to the target node. Notably, the pathological dysregulation of NFKB1, IFNB1, and IFNA1, which belong to the same feedback cycle (Figue S4), critically affects several driver-target functional interactions (Figure 5).

Finally, our approach allows us to identify a shift mechanism for dysregulated SOCS1 and SOCS3 drivers, showing opposite coactivation patterns in MS patients compared with the healthy controls (Figure 6). These results suggest that experimentally stimulating SOCS3—a strong inducer of pro-inflammatory response—might be more effective for moving the state of the altered secreted molecules towards physiological configurations. Taken together, these results might have practical consequences on how to design intervention strategies and counteract disease phenotype.

### Methodological Considerations

Our method uses Kalman controllability rank condition (Kalman, 1963) to quantify the centrality of the driver nodes. This criterion assumes that the investigated system has a linear dynamics (Equation 2). In our case, this means that the changes in the gene activation would follow a linear trend. While this is in general not true and difficult to ascertain, it appears that results from non-linear tests are often dominated by linear relationships (Song, Langfelder, & Horvath, 2012; Steuer, Kurths, Daub, Weise, & Selbig, 2002). Furthermore, a significant fraction of the data analysis and modeling deals exclusively with linear approaches as they are simpler and easy to interpret and serve as a prerequisite of nonlinear behavior (Y.-Y. Liu & Barabási, 2016).

Another peculiarity of our approach is the assumption of time-invariant interactions in the molecular gene network. On the one hand, this assumption allows us to better exploit the well-established results and tools in network controllability (Y.-Y. Liu et al., 2011); on the other hand, it might conflict with existing literature looking for biological connectivity changes between conditions or populations such as differential gene coexpression (Choi, Yu, Yoo, & Kim, 2005). Here, we hypothesized that the activation state of each node (in terms of gene expression) could eventually change, but not the underlying network structure. Thus, our network—obtained from detailed maps of the macrophage cells—would only act as a substrate/proxy for functional interactions, such as correlated gene activities.

Our method requires a specific ordering of the target nodes. While this can be typically achieved in many biological applications—by ranking nodes according to their state (e.g., gene expression: Liseron-Monfils, Olson, & Ware, 2018; Zhao, Yang, Huang, & Holme, 2011; brain activity: Lohmann et al., 2010; Sen, Chu, & Parhi, 2019)—challenges might still remain in general. When it is not possible to impose a ranking of the nodes from external knowledge, another possibility is to derive it from the network structure taking into account, for example, node centrality values. However, there might be multiple nodes with the same centrality value, which would impede a proper ranking. In those situations, multiple node centrality measures could be integrated to get a more heterogeneous distribution (e.g., degree, strength, betweenness, closeness; Newman, 2010). Another possibility is to add equally important targets at the same iteration step and test whether they are simultaneously controllable. While this procedure is suboptimal and may underestimate the number of controllable targets, it still minimizes the computational complexity related to testing multiple driver-target combinations.

We finally notice that our method is conceived for directed networks only, where the dimensionality reduction has a real computational benefit. In fact, in the case of undirected graphs, it is not possible to remove nodes on the walks from the driver to the targets since information is bound to span the entire network. Similarly, for directed but dense networks, the possibility to focus on specific parts of the network, and reduce the computational cost, becomes lower regardless of the topology.

To conclude, it is important to mention that extensions of network controllability tools to time-varying frameworks do exist (A. Li, Cornelius, Liu, Wang, & Barabási, 2016; Zhang, Garas, & Scholtes, 2017). However, in that case networks would be inferred from gene coexpression and therefore affected by statistical uncertainty due to sample sizes. Further research is needed to seek how to apply network controllability in presence of noisy time-varying connections.

### Conclusion

In this study, we introduce a method to quantify the ability of candidate driver nodes to drive the state of a target set within a sparse and directed network. Further, we illustrate how this method works for the molecular network associated with the human macrophage inflammatory response. The obtained results reveal in a principled way the genes that are significantly dysregulated in multiple sclerosis. We hope that this method can contribute to the identification of the key nodes in biological networks to better identify pharmacological targets to counteract human diseases.

## MATERIAL AND METHODS

### Stepwise Target Controllability

We introduce a method to identify which target nodes in a network can be controlled from a single *driver* node. To do so, we start by considering the canonical linear time-invariant dynamics on a directed network described by the adjacency matrix *A* ∈ ℝ^*N*×*N*^:

$$\dot{\text{x}}(n)=A\text{x}(n)-B\text{u}(n),\;\text{y}(n)=C\text{x}(n),$$ (2)

where **x**(*n*) ∈ ℝ^*N*^ describes the state of each node at time *t*, *B* ∈ ℝ^*N*^ specifies the driver node, **u**(*n*) ∈ ℝ^*N*^ is its external input (or control) signal, **y**(*n*) ∈ ℝ^*S*^ is the output vector, and *C* ∈ ℝ^*S*×*N*^ is the output matrix identifying the target nodes.

Such a system is controllable if it can be guided from any initial state to any desired final state in finite time, with a suitable choice of input. A necessary and sufficient condition to assess the controllability of Equation 2 is that the controllability matrix *Q*,

$$Q=\left[B\;AB\;A^{2}B\;\cdots \;A^{N-1}B\right],$$ (3)

has full row rank, that is, rank(*Q*) = *N*. That is the Kalman rank condition, which basically verifies the existence of linearly independent rows in *Q* (Kalman, 1963; Rugh & Kailath, 1995). If so, the driver node can reach and control the dynamics of all the other nodes through independent walks of length *N* − 1 at maximum.

If it is of interest to control only a target set 𝒯 of the network, specified in *C* and consisting of *S* ≤ *N* nodes, then Equation 2 can be reduced into a target controllability matrix *Q*_𝒯_ = *CQ* (Equation 1), where *C* filters the rows of interest corresponding to the targets. Now, the rank of *Q*_𝒯_ gives the number *τ* ≤ *S* of nodes in the target set that can be controlled by the driver.

To identify a driver-target configuration, we further introduce a hierarchy among the target nodes, so that we can order and relabel them from the most important one to the least, that is, *t*_1_ ≻ *t*_2_ ≻ … ≻ *t*_*S*_. Then we perform the following stepwise procedure for each candidate driver node:

- Step 1. *Initialization*
  –  Create a temporary empty target set 𝒯′ ← {}
  –  Set the number of controllable targets *τ* ← 0
- Step 2. *Repeat until termination criteria are met.* For *j* ← 1, …, *S* do
  –  Add the *j*-th target node to the target set 𝒯′ ← 𝒯′ ∪ {*t*_*j*_}
  –  Build the subgraph containing the nodes on walks from the driver to the targets in 𝒯′
  –  Compute the rank of the target controllability matrix *Q*_𝒯′_
  –  *If* rank(*Q*_𝒯_′) is full then *τ* ← *τ* + 1 else 𝒯′ ← 𝒯′ ∖ {*t*_*j*_}
  –  *j* ← *j* + 1
- Step 3. *Output* *τ* *and* 𝒯′

Eventually, the *target control centrality* *τ* is the number of controllable targets in 𝒯, and the set 𝒯′ contains the *τ* controllable targets with highest ranking.

Note that in general, the method may underestimate the actual number of controllable target nodes because of the occurrence of numerical errors, but not because of the stepwise procedure itself. The Matlab code associated with the stepwise target controllability is freely available at https://github.com/BCI-NET/Public.

### Construction of the Macrophage Activation Network

We reconstruct the inflammatory molecular network of the human macrophage by integrating information from the macrophage signal transduction map (Robert, Lu, Law, Freeman, & Hume, 2011; Raza et al., 2008). This map contains a comprehensive, validated, and annotated map of signal transduction pathways of inflammatory processes in macrophages based on the current literature. To extract molecular interactions from this map, we used the Hermit software (Motik, Cuenca Grau, & Sattler, 2008), which implements automatic reasoning based on logical rules. We specifically used the rules implemented in the molecular network ontology to infer molecular interactions depending on the process they belong (Henry et al., 2017; Musen, 2015). Because we are interested in the inflammation process, we restricted our analysis to a specific subset of 101 genes with known roles in macrophage pro-inflammatory activation, and for which their regulation in response to pro-inflammatory stimuli could be confirmed in our data set. These genes were classified according to their function in the cell: *sensing*, *signaling*, *transcription*, and *secreted* (Table S1), as described in databases such as NCBI Gene (Agarwala et al., 2018; UniProt Consortium, 2019), and GeneCards (Stelzer et al., 2016). The full network was thus reduced to include only these genes and their interactions. Because of recent studies, we also opted to exclude two edges (from SOCS3 to IFNGR1 and to IFNGR2) to represent the involved pathways (Wilson, 2014).

The resulting network contains *N* = 101 nodes and *L* = 211 unweighted directed edges representing either activation or inhibition between genes. The total degree *k* of each node in the network is computed by summing the number of incoming and outgoing edges:

$$k_{i}=\overset{N}{\underset{j=1}{\sum }}A_{ij}+\overset{N}{\underset{j=1}{\sum }}A_{ji},$$ (4)

where *A*_*ij*_ = 1 if there is an edge between the corresponding genes, and 0 otherwise.

### Collection of Macrophage mRNA Expression Data

Collection of blood for the study was approved by the French Ethics Committee and the French Ministry of Research (DC-2012-1535 and AC-2012-1536). Written informed consent was obtained from all study participants. All patients fulfilled diagnostic criteria for multiple sclerosis (Thompson et al., 2018), and individuals (multiple sclerosis patients and healthy donors) with any other inflammatory or neurological disorders were excluded from the study. Patients were included in the study only if they were not undergoing treatment.

Blood was sampled from eight MS patients and eight healthy controls in acid citrate dextrose tubes. From blood samples, peripheral blood mononuclear cells were isolated using Ficoll Paque Plus (www.gelifesciences.com) and centrifugation (2,200 rpm, 20 min). Cells were washed in PBS and RPMI + 10% FCS. Monocytes were isolated with anti-CD14 microbeads (www.miltenyibiotec.com) and plated in 12-well plates (500,000 cells/well) in RPMI + 10% FCS and granulocyte-macrophage colony-stimulating factor (500 U/ml) to induce differentiation into macrophages. After 72 hr, media was replaced with fresh media supplemented with granulocyte-macrophage colony-stimulating factor (500 U/ml) to maintain “alert” macrophages or IFN*γ* (200 U/ml) + upLPS (10 ng/ml) to induce “pro-inflammatory” activation. Cells were lysed after 24 hr and RNA was extracted with RNeasy Mini Kit (www.qiagen.com).

Transcriptome sequencing cDNA libraries were prepared using a stranded mRNA polyA selection (Truseq stranded mRNA kit, www.illumina.com). For each sample, we performed 60 million single-end, 75 base reads on a NextSeq 500 sequencer (www.illumina.com). RNA-Seq data analyses were performed by GenoSplice technology (www.genosplice.com). Sequencing, data quality, reads repartition (e.g., for potential ribosomal contamination), and insert size estimation were performed using FastQC (Andrews, 2010), Picard-Tools (http://broadinstitute.github.io/picard/), Samtools (H. Li et al., 2009) and rseqc (Wang, Wang, & Li, 2012). Reads were mapped using STARv2.4.0 (Dobin et al., 2013) on the hg19 Human genome assembly. Gene expression regulation study was performed (Noli, Capalbo, Ogilvie, Khalaf, & Ilic, 2015). Briefly, for each gene present in the FAST DB v2018_1 annotations, reads aligning on constitutive regions (that are not prone to alternative splicing) were counted. Based on these read counts, normalization was performed using DESeq2 (Love, Huber, & Anders, 2014) in R (v.3.2.5; R Core Team, 2014).

### Network Modeling and Data Analysis

In the modeling framework described by Equation 2, matrix *A* corresponds to the molecular network and represents the time-invariant component of the system. The dynamic component is instead represented by the gene activation response in the healthy and diseased condition (Figure 2B), computed as the ratio in gene expression between the “pro-inflammatory” and “alert” condition. Specifically, **x**(*n*) represents the gene activation. *B* is a vector identifying the candidate driver. The control signal **u**(*n*) is outside the scope of this work. The output vector **y**(*n*) and the output matrix *C* identify the target nodes.

We select the genes belonging to the *secreted* molecules class (Table S1) as our target set 𝒯. All the nodes in the other classes are then tested separately as potential driver nodes by computing their target control centrality *τ*. To enhance numerical precision, the logarithmic transformation log(*q* + 1) is applied to the elements of the target controllability matrix *Q*_𝒯_ (Equation 1).

The hierarchy among the target nodes is established by computing the fold change Δ between the corresponding gene activation in the two groups:

$$\Delta =\frac{\mu_{MS}}{\mu_{HC}},$$ (5)

where *μ*_*MS*_ and *μ*_*HC*_ are group-averages for MS patients and healthy controls, respectively, of the gene activation. Nodes with higher Δ absolute values are ranked first. Highly positive Δ values indicate a too strong inflammatory response (overactivation) in the MS patients with respect to the healthy controls. Highly negative Δ values indicate a too weak inflammatory response (underactivation). We define *dysregulated* genes along the controllable driver-target walks as those for which |Δ| is above the 75th percentile.

We perform a robustness analysis to evaluate the stability of the identified driver nodes to potential errors in the molecular network reconstruction. We simulate attacks with increasing intensity, that is, up to 20% of the nodes or edges in the network. When removing nodes, we consider the following cases: (a) random deletion, (b) preferential removal of high-degree nodes, and (c) preferential removal of low-degree nodes. Preferential attacks are performed by selecting nodes with a probability *p* proportional to their degree *k*, that is, *p* ∝ *k* for high-degree nodes and *p* ∝ −*k* for low-degree nodes. When perturbing edges, we test: (a) random addition, (b) random deletion, and (c) random rewiring. For each case, we simulated 1,000 repetitions and we computed the target control centrality *τ* for the driver nodes identified in the original network. Then, we report the percentage of nodes that cease to be drivers (i.e., *τ* = 0), that is, the percentage of nodes that are drivers in our analysis, but are no longer able to control any target in the perturbed case.

## ACKNOWLEDGMENTS

We would like to thank Professor Albert-Laszlo Barabasi for his helpful comments and suggestions. The content is solely the responsibility of the authors and does not necessarily represent the official views of any of the funding agencies.

## SUPPORTING INFORMATION

Supporting information for this article is available at https://doi.org/10.1162/netn_a_00180.

## AUTHOR CONTRIBUTIONS

Giulia Bassignana: Conceptualization; Data curation; Investigation; Methodology; Software; Validation; Visualization; Writing – original draft; Writing – review & editing. Jennifer Fransson: Data curation; Methodology; Writing – original draft; Writing – review & editing. Vincent Henry: Data curation; Formal analysis; Methodology; Software; Writing – original draft; Writing – review & editing. Olivier Colliot: Conceptualization; Funding acquisition; Project administration; Supervision; Writing – original draft; Writing – review & editing. Violetta Zujovic: Conceptualization; Data curation; Funding acquisition; Methodology; Project administration; Resources; Supervision; Writing – original draft; Writing – review & editing. Fabrizio De Vico Fallani: Conceptualization; Investigation; Methodology; Supervision; Validation; Visualization; Writing – original draft; Writing – review & editing.

## FUNDING INFORMATION

Olivier Colliot, Agence Nationale de la Recherche (http://dx.doi.org/10.13039/501100001665), Award ID: ANR-19-P3IA-0001. Olivier Colliot, Agence Nationale de la Recherche (http://dx.doi.org/10.13039/501100001665), Award ID: ANR-10-IAIHU-06. Olivier Colliot, Inria, Award ID: Project Neuromarkers.

## TECHNICAL TERMS

Controllability: Ability to act upon a network for steering its state towards desired configurations.
Graph: Set of nodes and edges representing the units and the links of a network.
Driver node: Node of the network candidate to steer the target sets state.
Target set: Subset of nodes in the network that are chosen to be controlled.
Walk: Alternating sequence of nodes and edges in a network.
Fold change: Ratio between the gene expression of diseased and healthy states.
Node degree: Number of links of a node.
