# Supplementary material for: Stepwise target controllability identifies dysregulations of macrophage networks in multiple sclerosis
Source: Netw Neurosci. 2021 Apr 27;5(2):337–57. doi: 10.1162/netn_a_00180 (PMC8233109; doi:10.1162/netn_a_00180)
Supplement: Supplementary file 1 [file netn-05-337-s001.pdf]

Bassignana, G., Fransson, J., Henry, V., Colliot, O., Zujovic, V., & de Vico Fallani, F. (2020). Supporting information for "Step-wise target controllability identifies dysregulations of macrophage networks in multiple sclerosis." *Network Neuroscience.* Advance publication. https://doi.org/10.1162/netn_a_00180

Supplementary figures

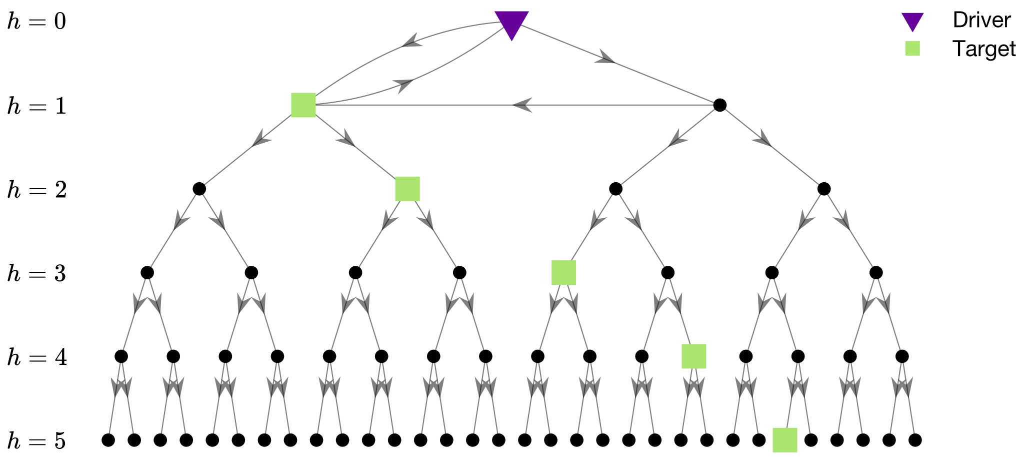

Fig S1. Methodological validation of step-wise target controllability. We start from a simple directed full binary tree, and we add a cycle among the first three nodes. The driver is the root of the tree, while we put a target node in each level of the tree. Target nodes are then ranked according to their height h. This configuration is fully controllable by construction regardless of the tree's height h. However, for $h = 5$ (i.e. $N = 63$), the standard procedure computing the rank of the full Kalman controllability matrix cannot retrieve all the targets, as the rank computation is deficient due to numerical errors. Instead, by using the step-wise target controllability we can correctly identify the controllable targets up to $h = 10$, i.e. $N = 2047$.

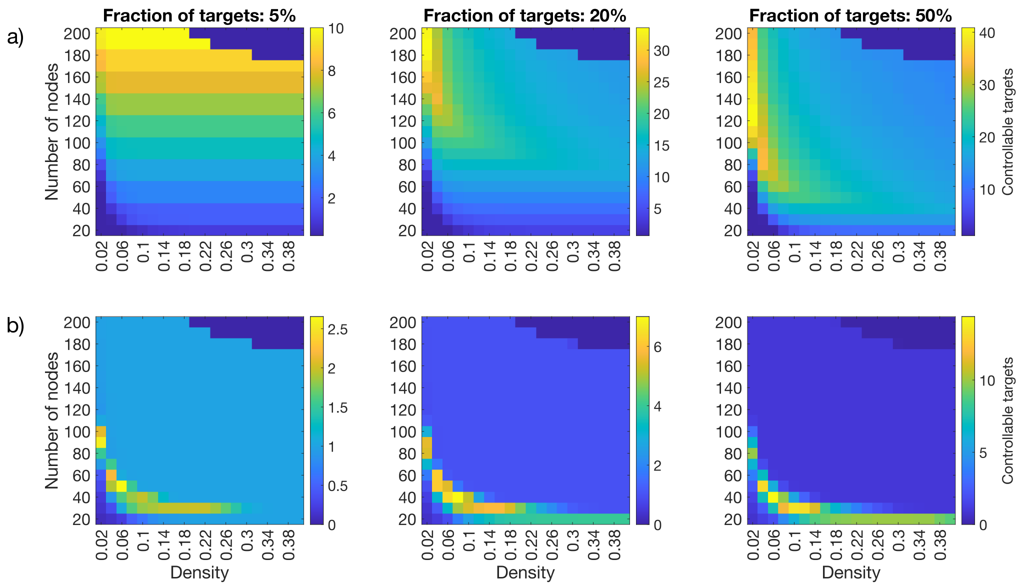

Fig S2. Average number of targets that can be controlled by a single driver node, computed on samples of 100 connected and directed random networks, at the varying of size and density of the networks, and target set size. Panel **a)** corresponds to the step-wise target controllability. Panel **b)** corresponds to a standard procedure that computes the rank of the full Kalman controllability matrix. Results show that in general our method is able to retrieve a larger number of controllable targets as compared to the standard procedure. More specifically, when the target set contains 5% of the network nodes, results are quite stable across different connection densities. For larger target-set sizes our method works better when the connection density is relatively low (0.02-0.10). It is important also to notice that the computation of the rank starts to fail in correspondence of larger and denser networks (i.e., $N > 180$ and density > 0.20).

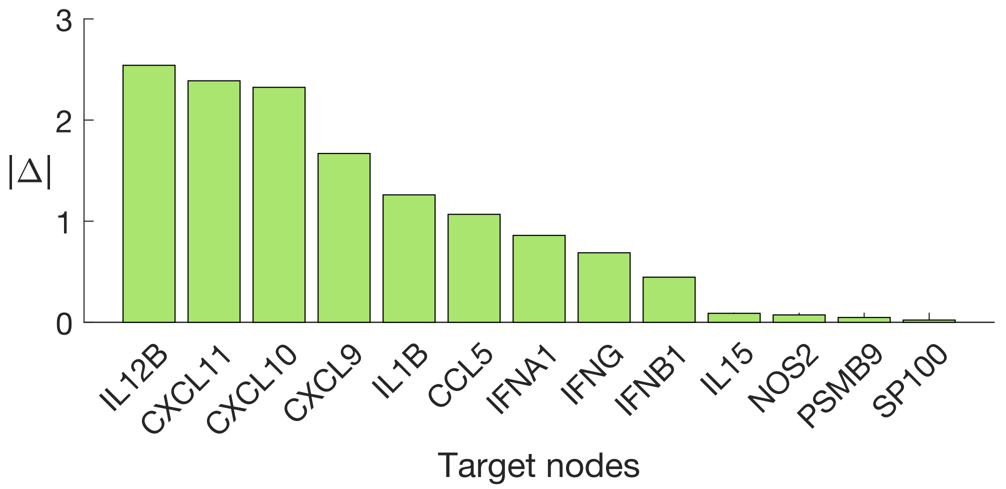

Fig S3. Hierarchy among target genes. Genes corresponding to the 13 secreted molecules are ranked according to the absolute value of fold change Δ in the gene activation between the multiple sclerosis (MS) group and the healthy control (HC) group (**Materials and methods**).

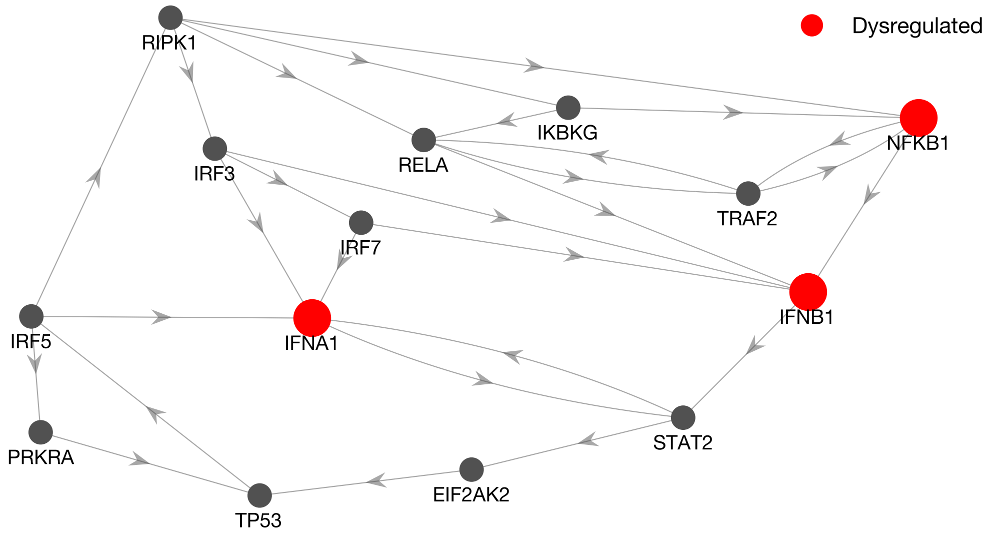

Fig S4. Subnetwork illustrating the feedback cycle between dysregulated genes IFNA1, IFNB1 and NFKB1. The three nodes belong to the only strongly connected component (a subnetwork in which every node is reachable from any other node) of the network having more than two nodes. It plays, thus, a central role in the network topology.

Captions of supplementary tables

Table 1. List of all node genes and associated class depending on their functional role.

Table 2. Distribution of driver nodes across different classes of genes.

Table 3. Spearman cross-correlation and p-values values for controllable driver-target node pairs. Values for pairs that were not controllable were replaced by not-a-number (NaN). Both values for multiple sclerosis patients (MS) and healthy controls (HC) are reported.

Table 4. Log-transformed gene expression for multiple sclerosis patients (MS) and healthy controls (HC), in the 'alert' (M0) and 'pro-infalammatory' (M1) macrophage activation states; computation of the Δ as fold change.
